# Intermittence and connectivity of interactions in pigeon flock flights

**DOI:** 10.1038/s41598-017-09986-5

**Published:** 2017-09-05

**Authors:** Duxin Chen, Xiaolu Liu, Bowen Xu, Hai-Tao Zhang

**Affiliations:** 1 Guangdong HUST Industrial Technology Research Institute, Guangdong Province Key Lab of Digital Manufacturing Equipment, Dongguan, 523000 China; 2 Key Laboratory of Image Processing and Intelligent Control, School of Automation, State Key Laboratory of Digital Manufacturing Equipments and Technology, Huazhong University of Science and Technology, Wuhan, 430074 China; 3 Department of Biological Physics, Eötvös Loránd University, Budapest, H-1117 Hungary

## Abstract

Collective circular motion is a common yet spectacular behavior of pigeon flocks. Efficient and robust inter-individual communication is required for flock coordination during this widely-spreaded movement pattern. When a flock hovers near the home loft, the rotational direction undergoes regular spontaneous variations. Based on intensive analyses of high spatial-temporal resolution GPS data, we quantified the intensity of velocity alignment among different individuals in terms of their velocity fluctuations. It is found that pigeon flocks employ an intermittent interaction (alignment) mechanism, where intra-group information transmission is not required at every instant. However, the union of the topologies of several consecutive interaction networks always keeps connected. This biological observation strongly suggests the presence of a jointly connected topology in pigeon flocks, which helps substantially reduce the communication and/or information processing requirements while retaining the agility and stability of the group. Finally, we conducted extensive investigation on the interaction mechanism as well as the spontaneous changes in rotational direction of pigeon flocks. These results shed some light onto the coordination mechanism exploration of bird flocks’ collective motions.

## Introduction

In nature, cooperative behavior is common in biological systems ranging from microscopic to macroscopic levels, such as bacterial colonies^1^, migrating cells^2^, insect swarms^3^, fish schools^4^, bird flocks^5^ and mammal herds^6–8^. To understand the often encountered spectacular behavior of social animals, collective motion analysis has been widely carried out in recent years, and it is continuing to attract more and more attention from biologists, as well as physicists, life scientists, and computer scientists^9^. As a milestone study, Vicsek *et al*. proposed a well-known flock model, where each agent’s direction of movement is determined by the average direction of its neighbors. The so-called Vicsek model (VM)^10^ captures the behavior of highly ordered structures that emerge in bird flocks. Another well-known study^11^ of Couzin *et al*. proposed a three-sphere model that yields three typical patterns of universal collective motions in fish schools, i.e., swarm, torus, and migration states.

Recently, Ballerini *et al*.^12^ proposed a new alternative possibility for the interaction rule in a huge flock comprising about 2600 starlings, where it was observed that each bird interacted with only a fixed number of topological neighbors, instead of individuals within a specific metric distance. This model provided a better explanation of the interaction mechanism among starlings, which was later reinforced by both theoretical analysis^13^ and inter-species experiments in mosquitofish schools^14^. By further considering visual sensory limitation, Strandburg-Peshkin *et al*.^15^ predicted the propagation of behavioral change in fish schools during leadership events, and found that structural properties of visual interaction networks differ remarkably from previous relative spatial position-based models, including metric^10^ and topological^12^ counterparts, which expanded the understanding of collective flocking behaviors. Using a GPS tracking device, Dell’ Ariccia *et al*.^16^ studied homing pigeons (*Columba livia*) and found that the homing performance of birds flying in a flock was significantly better than that of birds released individually. Still, using high-resolution GPS data obtained from pigeon flocks, a hierarchical leadership network was revealed by Nagy *et al*.^17^, where each pigeon acts as a leader or a follower, or plays a dual role when situating on middle layers. To investigate whether pigeon flocks obey a hierarchical or egalitarian interaction pattern, Zhang *et al*.^18^ explored free flights of pigeon flocks and indicated that each pigeon tends to follow the average of its neighbors while moving along a smooth trajectory, whereas it switches to follow the leaders upon sudden turns or zigzags occur. Later, Chen *et al*.^19^ reanalyzed the same homing flight datasets^17^ and indicated that a pigeon flock has a fixed long-term leader for smooth moving trajectories in homing flights, whereas the leadership passes to a temporary one on sudden turns or zigzags. To investigate the principle governing self-organized patterns, Ferrante *et al*.^20^ developed an active-elastic-sheet, which well explains the emergence of the ordered state of both natural and artificial swarms. Based on stereo imaging techniques, Attanasi *et al*.^21^ collected a high-resolution spatial dataset composed of thousands of starlings, which they used to formulate a realistic flocking dynamics model concerning spontaneous symmetry breaking and conservation rules. This model suggests that turning information propagates across the flock according to a linear dispersion law with negligible attenuation. Aforementioned studies have provided specific insights into industrial applications of a huge volume of networked processes or multi-agent systems, such as unmanned air vehicles^22^, attitude alignment of satellite clusters^23^, and multi-robot formation control^24, 25^. Still, from mathematical analytical point of view, a general theoretical framework describing the dynamics of biological group behaviors was presented by Olfati-Saber *et al*.^26^, which provides deep insights into the emergence of highly-coordinated group behavior by simple inter-individual interactions. Jadbabaie *et al*.^27^ suggested a connectivity condition to guarantee that no agent escapes from the influence of the entire group.

Previous studies^28–31^ have shown that group decision-making strategies can not be elucidated without deeply understanding the interactions among individuals. Thus, an increasing number of investigations have been devoted to the intra-group interactions^32^, communication networks^33^, and information transmission mechanisms^15^. However, the influence of inter-agent connection on bird flock dynamics is still far from being fully understood. For instance, do pigeons interact with others all the time? Are the interaction intensities among pigeons flocks strong or weak? To answer these fascinating questions, we focused on circular movements and conducted a detailed analysis on combined datasets consisting of 41 releases of four pigeon flocks, each of which has ten individuals. We used the data of three pigeon flocks (labeled here as in the original paper: flocks *A*, *B*, and *C*) with 30 releases from^34^ (sampling period 0.1 s), as well as one flock (labeled here flock *D*) with 11 releases (labeled ff1-ff11 free flights) from ref. 17 (sampling period 0.2 s). We observed that, during the flight process, a pigeon did not align itself with others frequently, but only occasionally. This biological observation strongly suggests that pigeon flocks adopt an intermittent alignment mechanism. More significantly, it implies that, although the interaction (i.e., alignment) network of each instant is not necessarily connected, their union always keeps connected. It should be noted that throughout the study, we only focus on the ordered flock state during free flights, interaction is thus deemed as the widely-used index of pairwise velocity alignment^17, 35, 43^. Therefore, the present study gives an biological clue of the existence of jointly connected interaction networks in pigeon flocks.

## Results

### Intermittent interaction mechanism

We focus on the emergence of a highly ordered state during the free flights of pigeons. The degree of ordering in a flock is measured by the so-called order parameter $\varphi \,\mathrm{=1/}N\parallel {\sum }_{i=1}^{N}{\overrightarrow{v}}_{i}(t)/||{\overrightarrow{v}}_{i}(t)\parallel$, which is employed as a standard index of order during the study of collective animal behavior^10^. Here, ${\overrightarrow{v}}_{i}(t)$ denotes the velocity of bird *i* in the horizontal plane, and *N* is the total number of birds in the flock. Apparently, the index *ϕ* is zero for totally disordered and one for synchronized flocks (the evolution of the index *ϕ* for flock *D* in the ff2 release is shown in Figure S1).

**Figure 1:**
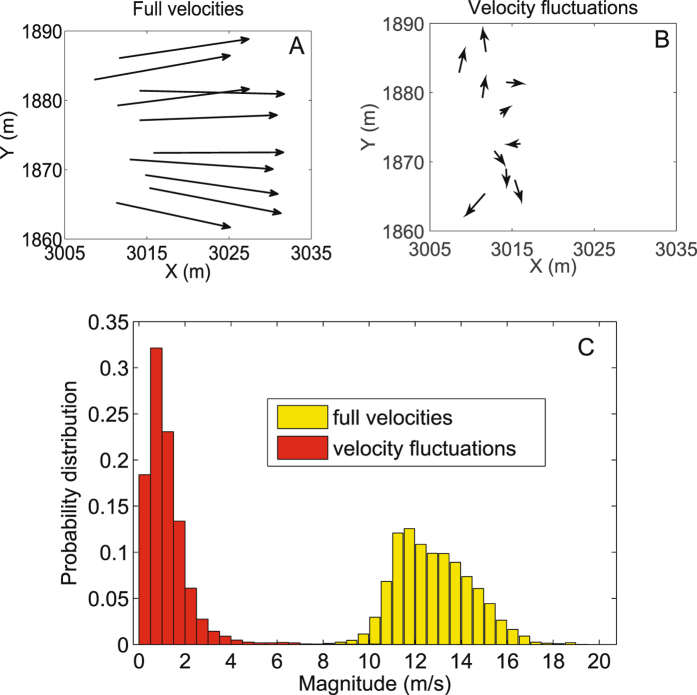
(**A**) Temporary individual velocities, which are almost synchronized. (**B**) The fluctuations of individual velocities at a same instant. (**C**) Normalized probability distribution of the modulus of velocities and velocity fluctuations. The modulus of velocity fluctuations are much smaller than those of the velocities. Thus, velocity fluctuation provides a sharper index for measuring the pairwise correlations, especially for highly synchronous collective motions.

**Figure 2:**
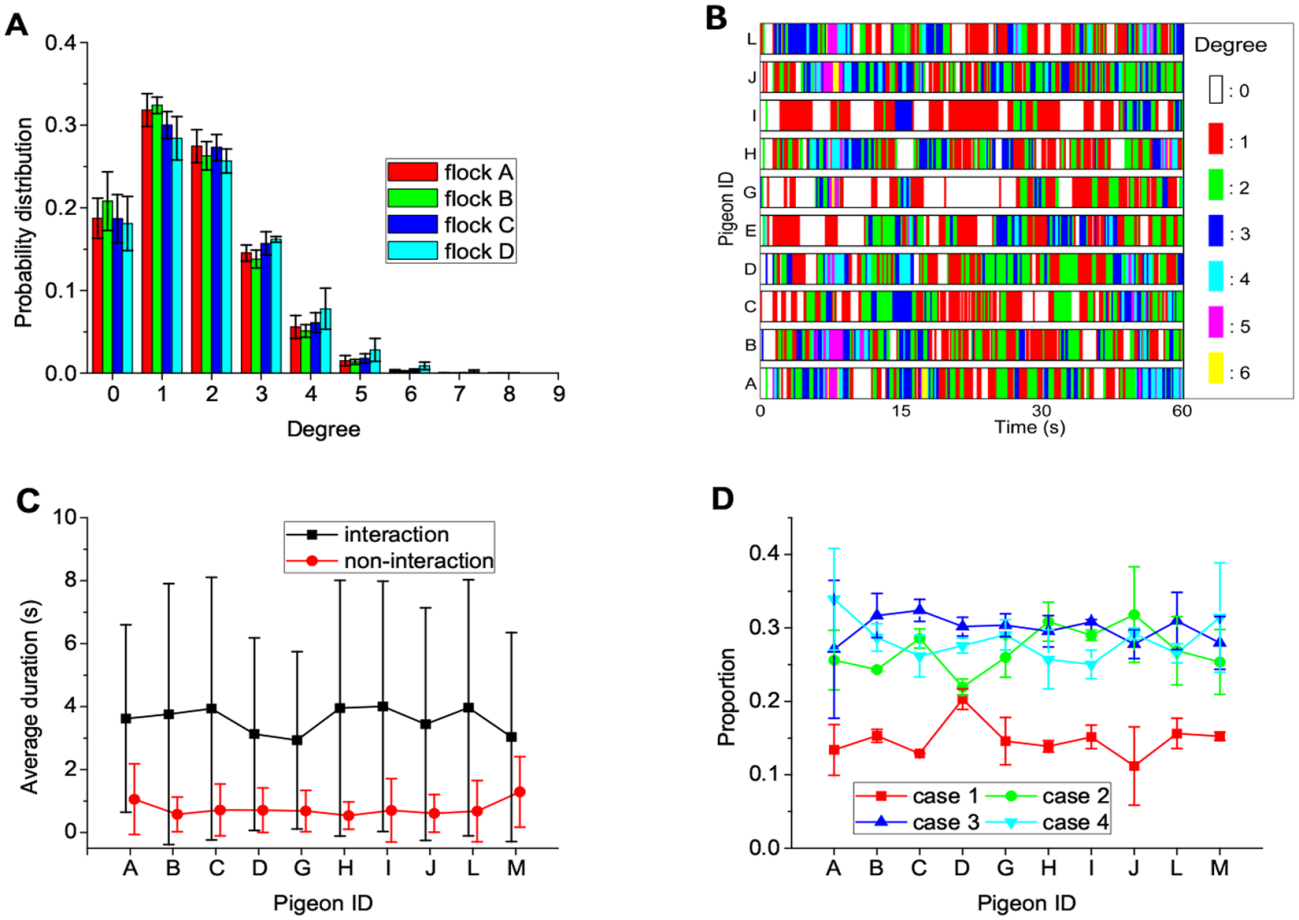
(**A**) Degree distribution of the pigeon flocks. For each flock, individuals typically interact with one or two neighbors and do not interact globally with all the members. (**B**) Illustration of the intermittent interaction mechanism. Different colors represent different numbers of neighbors for each individual. (**C**) Average durations of both interaction and non-interaction situations corresponding to each pigeon. Error bars indicate the standard deviations. For each individual, the average durations of both interaction and non-interaction situations remain steady in free flights. (**D**) Proportions of the four cases in flock *D*. *Case 1:* the pigeon only interacts with its neighbors without time delay. *Case 2:* the pigeon has directed interactions and plays the dual role as a leader and a follower simultaneously. *Case 3:* the pigeon has directed interactions and act as a follower. *Case 4:* the pigeon has directed interactions and plays the role as a leader. It is observed that the case when individuals only have undirected interactions without time delay occupies a smaller proportion, whereas directed interactions with time delay are more frequently encountered cases in pigeon flocks.

In order to quantify the intensity of velocity alignment between each pair of individuals, we formulate a quantity related to the instantaneous correlation with time delay of two pigeons (see Methods) in terms of their velocity fluctuations (Fig. 1A–C). Since the magnitude of velocity fluctuation is much smaller than that of full velocity, it provides a sharper index for measuring the pairwise correlations, especially for highly synchronous collective motions. A previous study^35^ proposed a minimally structured (maximum entropy) model to investigate the interaction mechanism in large flocks of starlings, where the pairwise strength of interaction is defined as the intensity that the pair tend to align their moving directions. However, in small-sized pigeon flocks, we use the velocity fluctuation vector (both magnitude and direction are considered) to figure out the specific interactions in circular motions. In detail, the two individuals are considered to interact with each other when the instantaneous correlation (Eq. (1)) quantifying the velocity alignment intensity has a sufficient large value. Noted that individuals interact with each other by previously perceived information^17^, therefore, the index *τ* representing the time delay has been considered in the definition. After analyzing the combined datasets^17, 34^, the degree (number of neighbors) distributions are shown in Fig. 2A. It is observed that the mostly encountered case is that individuals merely interact with one or two neighbors. In addition, no pigeon interacts with all the other members in the flock at each time instant. This also suggests that the information achieved by one pigeon only propagates to a few neighbors at one instant.

According to Fig. 2A, we can predict that only the local diffusive transmission of information propagates among pigeon flocks. To investigate the dynamic characteristics of each individual, we also show the evolutionary interaction of each pigeon in Fig. 2B. In order to demonstrate the pairwise interactions more vividly, we show slots with different colors which indicate different numbers of communicating pigeons (i.e., neighbors) at a specific instant. Clearly, white and colorful regions appear intermittently, which suggests that each pigeon adopt an intermittent interaction protocol during the whole flight. In other words, some independent individuals may not interact with others, but spontaneously join the interaction now and then. We show the average durations of both interaction and non-interaction situations corresponding to each pigeon of flock *D* in Fig. 2C (other flocks, Figure S2). It is observed that each pigeon maintains the interaction with neighbors during around 3.5 s, and flies independently for some subsequent instants. Furthermore, the average durations of both interaction and non-interaction situations remain steady. Such observation indicates the reduced energy of communication and/or information processing defined as the proportion of the time that a pigeon does not interact with others. To examine the generality of the observation for different sampling periods, we also show the results of 5 Hz frequency case for flocks *A*, *B* and *C* in Figure S3, respectively. The results keep consistent with those of sampling period 0.1 s, which validates that sampling time does not influence the results. With a time delay, the instantaneous correlation (Eq. (1)) includes both non-interaction and interaction conditions. More interestingly, four distinct interaction cases can be formulated as well, such as *Case 1:* the pigeon only interacts with its neighbors without time delay; *Case 2:* the pigeon has directed interactions (with time delay) and plays the dual role as a leader and a follower simultaneously; *Case 3:* the pigeon has directed interactions and only act as a follower; *Case 4:* the pigeon has directed interactions and only plays the leading role. The proportions of the four cases in flock *D* are shown in Fig. 2D. It is observed that directed interactions with time delay are more frequently encountered than undirected interactions (other flocks, Figure S4).

### Jointly connected network

**Figure 3:**
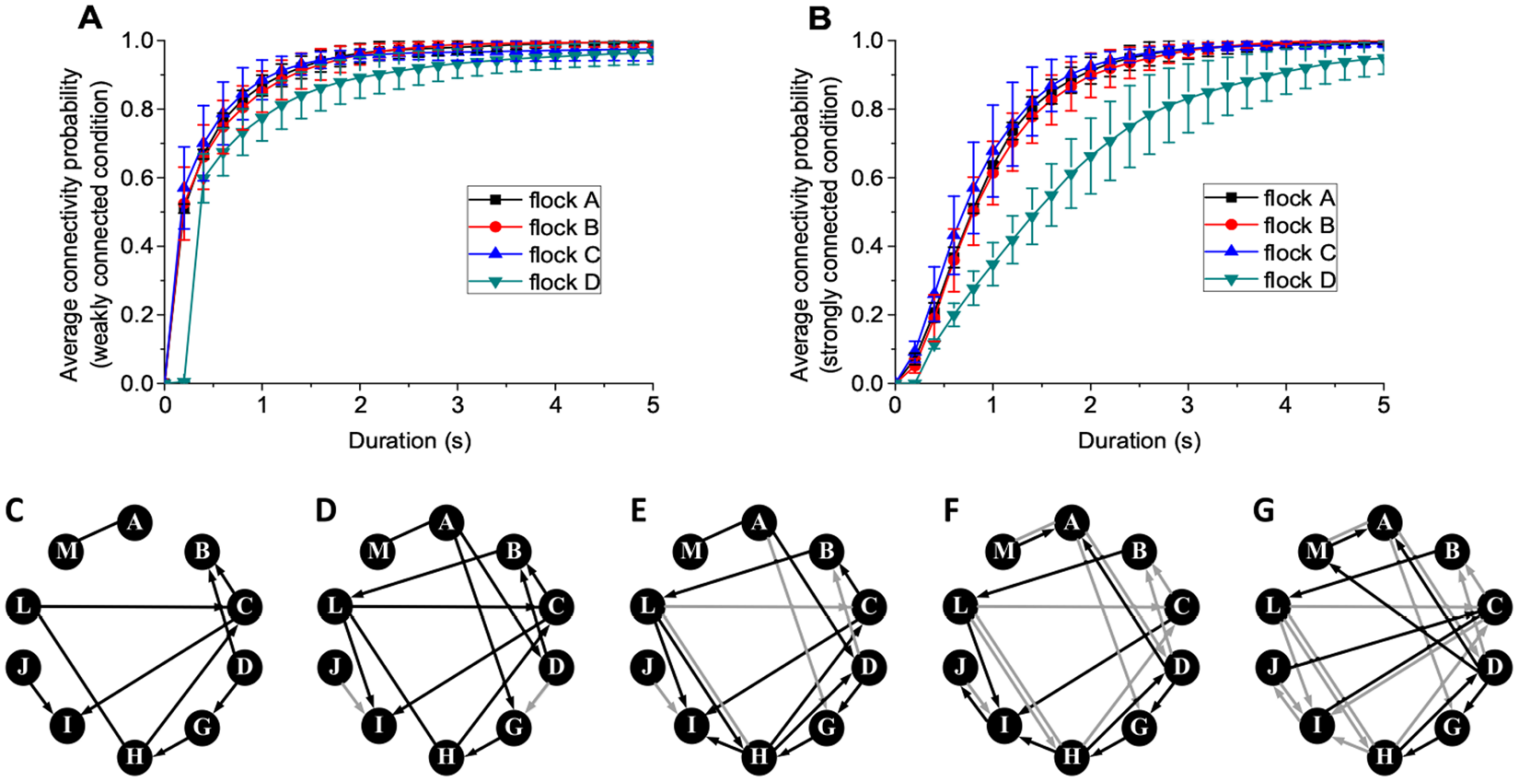
Connectivity probability of the interaction networks. (**A**) Weakly connected condition. (**B**) Strongly connected condition. Each flock consists of 10 individuals. Durations indicate the number of continuous instants of interaction networks merging into a union. Connectivity probability is calculated as the proportion of connected situations. A small possibility of connectivity corresponds to small durations of interaction networks, whereas it grows large with an increasing duration. (**C**–**G**) Networks of five consecutive time instants (recorded with 0.2 s sampling rate) in release ff11 of flock *D*. Black lines indicate the present connections, whereas grey ones the connections accumulated from previous time steps. A straight line (without arrow head) shows that the two individuals interact synchronously, whereas the arrow pointing from pigeon *i* to pigeon *j* indicates that *i* lags behind *j* with a positive time delay. By merging two consecutive networks together, a weakly connected graph emerges. Note that only the network corresponding to the union of the five instants is strongly connected.

The instantaneous correlation (Eq. (1)) defines a directed relationship for non-zero time delays for each pair of pigeons. A directed graph is called weakly connected if replacing all the directed edges with undirected edges produces a connected undirected graph. The term *strongly connected* is used if the network contains both a directed path from *u* to *v* and the reverse for every pair of nodes *u* and *v*. In this paper, both the weakly and strongly connected conditions of interaction networks are investigated. As shown in Fig. 3A,B, a small possibility of connectivity is associated to the union of interaction networks at some consecutive instants. Evidently, the possibility grows larger with increasing durations. If the flock is more hierarchical, transitive relationships are more directed with fewer feedbacks from the lower level to the higher level, which results in a smaller possibility for the flock to achieve a strongly connected topology. Thus, under both the weakly and the strongly connected conditions of the directed networks, flock *D* (trained racing pigeons, more hierarchical, flying above urban area in Budapest) has lower connectivity probability compared with those of flocks *A*, *B*, *C* (free-ranging domestic pigeons, flying above country area near Oxford). It indicates that pigeon flocks employ a jointly connected interaction network in free flight where the instantaneous interaction network does not keep connected, but the union of several continuous instants becomes connected. If we consider a sufficiently long period, a path will always exist in the union of the sequential instantaneous interaction networks from one individual to any other in the flock. It should be noted that the network represents a whole landscape of pairwise velocity alignments, but not the channels of information transmission. Although in small bird flocks, information may independently transfer extremely swiftly^21^, individuals do not prefer to interact at every instant. To give a clearer illustration of the evolution of connectivity, we pick the networks of five consecutive instants in flock *D*, which are shown in Fig. 3C–G. It is weakly connected when mergeing two consecutive networks into a union. However, only the network corresponding to the union of the five instants shown in Fig. 3G is strongly connected. Compared to weakly connected conditions, a strongly connected scenario is rarer to be achieved. Now a question is naturally inspired: to achieve coordination, whether pigeon flocks employ a strongly or weakly connected mechanism? In rotational movements, we suggest that a jointly and weakly connected condition is essential, whereas strong connections improve both extensiveness of information transmission and coordination among individuals.

**Figure 4:**
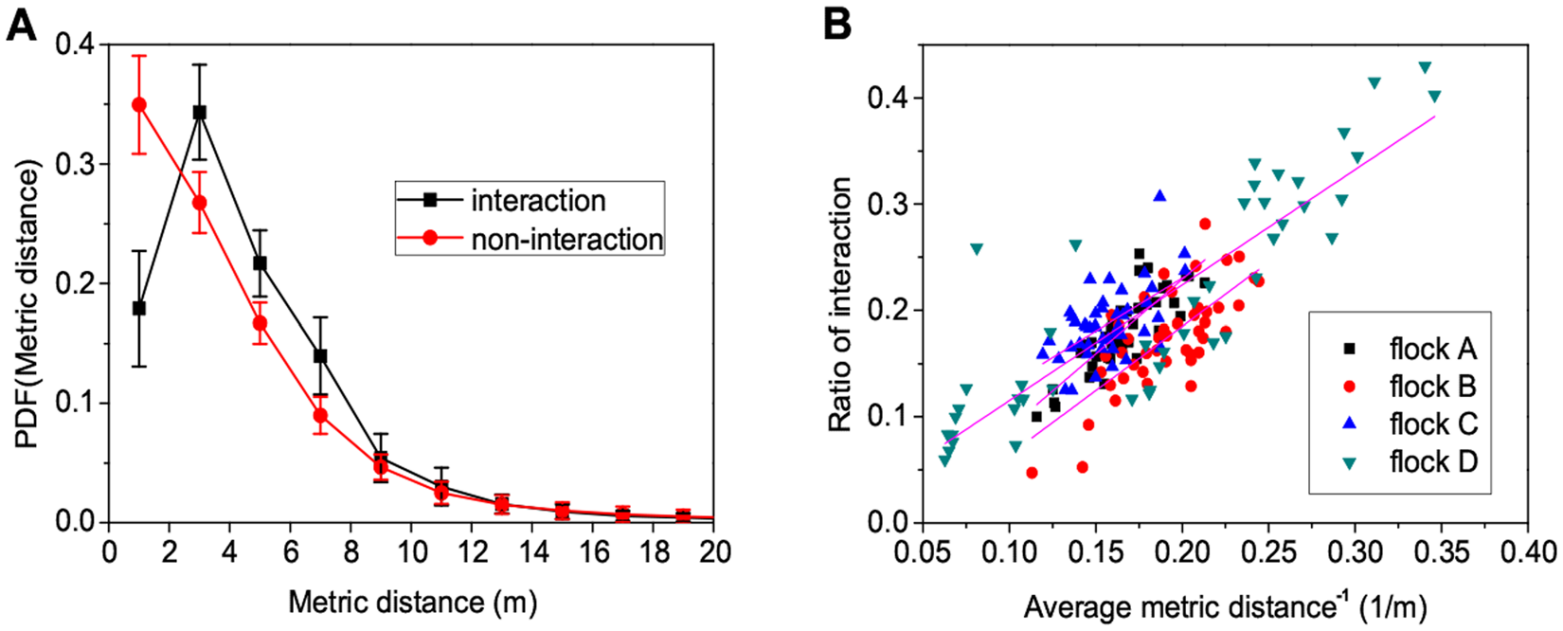
(**A**) Probability distribution of pairwise metric distances corresponding to both interaction and non-interaction. The probability distribution of non-interaction decays strictly with increasing pairwise metric distances, whereas for interaction situation, the maximum occurs within the range of 2–4 m. (**B**) Scatter plot for all possible pairs (45 pairs from 10 individuals) of each flock showing the relationship between the ratio of interaction and the reciprocal of average metric distance. Significant correlation is observed for all flocks.

To investigate the pairwise interaction among individuals, we show the heat map of interaction occurrence rate of each pairwise members in Figure S5 (four releases of flock D). During the entire flights, almost all members may have pairwise interaction among the flock. However, for a same flock, the interaction occurrence rate of each pairwise members is not fixed in different releases. In addition, we show the probability distribution of pairwise metric distances of both interaction and non-interaction situations in Fig. 4A. It is observed that the probability distribution of non-interaction decays strictly with increasing pairwise metric distances, whereas for interaction situation, the maximum occurs within the range of 2–4 m. Substantially, pigeons interact more frequently to their neighbors with smaller metric distance. Meanwhile, we show the scatter diagram with linear fitting lines for all pairs of ratios of interaction and the reciprocals of average pairwise metric distance in Fig. 4B. A significant positive correlation is observed by using permutation tests on each individual (nine pairs, sampling size: *n*_1_ = *n*_2_ = 9) in four flocks *A*, *B*, *C*, and *D* (mean ± SD: *p*_*A*_ = 0.0052 ± 0.0062, *p*_*B*_ = 0.0052 ± 0.0062, *p*_*C*_ = 0.0052 ± 0.0062, *p*_*D*_ = 0.0044 ± 0.0060).

### Theoretical model

To quantitatively verify the understanding of circular motions with spontaneous changes in rotational directions, we propose a self-propelled particle model based on an intermittent interaction mechanism. Therein, three types of forces act on each particle: centripetal, alignment, and homing forces (Figure S6 and Methods). The centripetal force is designed to drive each individual to rotate. Meanwhile, we use the self-dynamics Eq. (3) (Methods) to generate the independent circular motion for each individual. The alignment force is defined as the combination of neighboring forces to achieve the convergence of the circular centers. The homing force is used to yield attraction from the loft to each pigeon, which is designed as a convex function. With respect to homing force, a previous study^36^ has reported the tendency of pigeons towards the roosting area and introduced three types of forces to simulate the free flights, i.e., homing force, velocity regulation, and interaction/alighment force. The motions of individuals are mediated by the positions, velocities, and directions of their neighbors, as assumed in the VM^10^. In addition, we employ a time-varying connectivity topology and apply a restriction on the communication capacity of each individual. More precisely, for each individual, we randomly pick five individuals as its neighbors if there are more than five ones within its interaction range. Note that if the connections are stronger with larger neighborhood size, then collective motion is more rapidly achieved^37^. But this implies greater communication cost. Therefore, the proposed model with limited communication capacity enables the verification of the feasibility of the inferred rules for reproducing coordinated circular motions. Additionally, the anisotropy of interaction based on our correlation method has been considered (Figure S7). Comparing with the results in starling flocks^12^, we observe that pigeons tend to interact with neighbors located right or left, but not along their velocity directions. To focus on the more crucial mechanism of intermittent interaction, we have not introduced the non-linear function related to the anisotropic visual apparatus of birds into the alignment force.

To simulate and explain how a heterogeneous flock of pigeons can achieve spontaneous changes in rotational direction, we assume that every individual has a unique depletion time^38^ which follows a Gaussian distribution ${t}_{i}\sim N(\mu ,\sigma )$ (see Table 1 for the parameters) without explicit interaction of inner variables (see Figure S8 and Methods). More precisely, each pigeon is assigned a priori value of the length of flight time *t*_*i*_, at which the pigeon will start to feel the depletion of its energy reserve, and would be increasingly wishing to change rotational direction. When a pigeon feels “tired” or wants to change the rotational direction but the others not, it must follow them unwillingly but with increasingly greater intension to change^39^. Thus, when a sufficient number of members have been accumulated willing to change their rotational direction, they drive the whole flock to switch. More precisely, when individual *i* reaches its depletion time *t*_*i*_, the noise bias of angular speed increases from 10% to 20% to facilitate the rotational change. When the directional difference between the biased individual and the mean of the flock reaches 45°, it will drive the entire flock to switch.

**Table 1:** Parameters used in the pigeon flock data analysis. We select *R* = 1, *D* = 1, and Δ*t* = 1 to be the normalized length, angle, and time, respectively.

Parameter	*ν*_0_	*ω*_*i*_	*U*_*A*_	*U*_*B*_	*ρ*_*ƒ*_	*c*_0_	*w*_*α*_	*w*_*β*_	*L*	*μ*	*σ*	*ξ*
Value	20	0.5	100	20	5	2.5*e* ^−6^	0.2	2.2	400	250	30	2
Dimension	*R*/Δ*t*	*D*/Δ*t*	*R*	*R*	*R*/Δ*t*	—	—	—	*R*	Δ*t*	Δ*t*	*R*/Δ*t*

**Figure 5:**
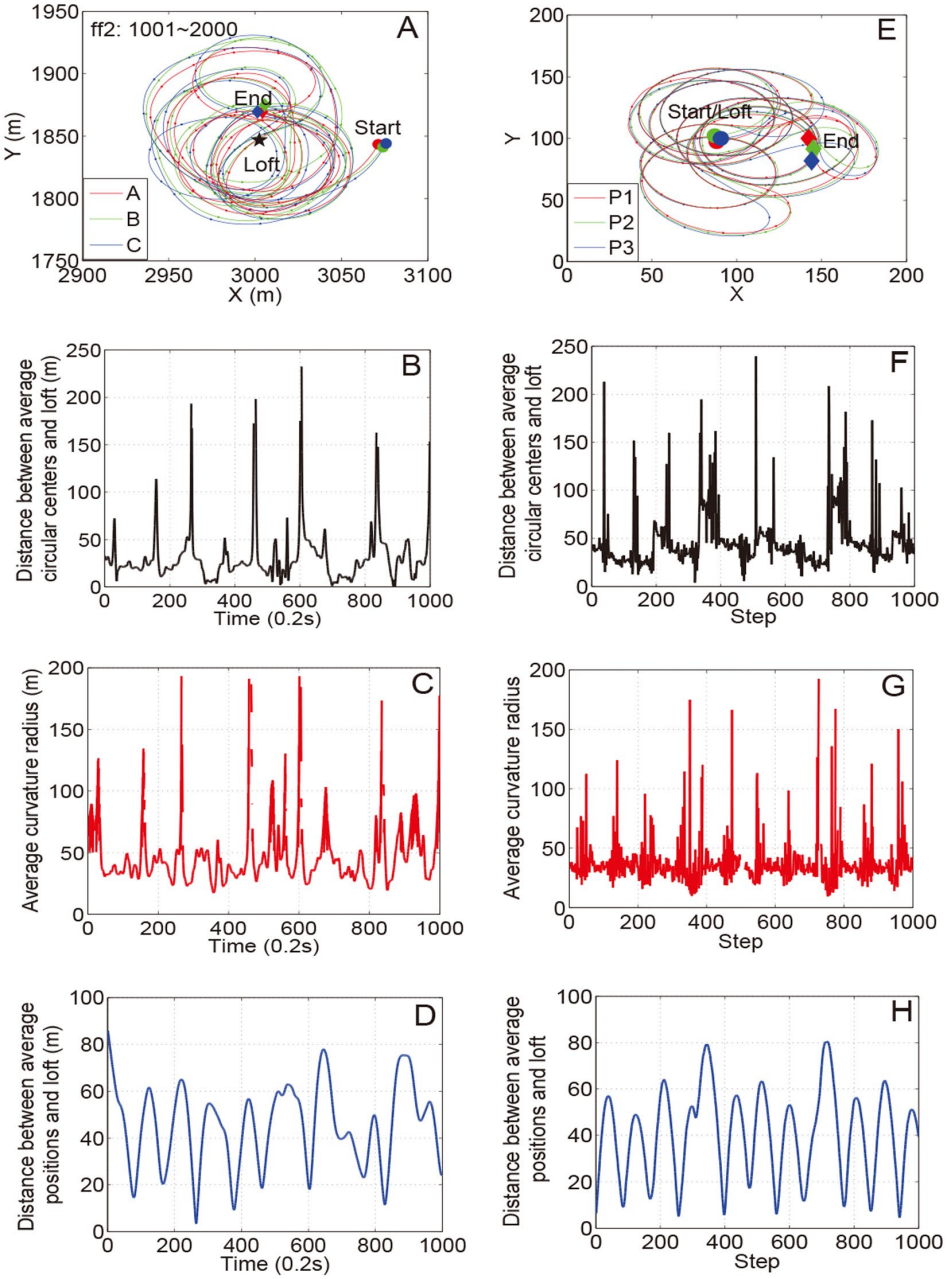
Comparison between experimental results and numerical simulations. (**A**) 200 s long (1000 time steps) segment of flight trajectories from flock *D* release ff2. For better visibility, only three pigeons are exhibited. (**B**–**D**) The distances between the average circular centers and loft, average curvature radius, and distances between the average positions and loft, respectively. If the pigeon flock flies relatively straight for three continuous instants, the average curvature radius will grow larger in synchronized manner with peaks in (**B**) and (**C**). (**E**–**H**) Results corresponding to the 1000 steps of the numerical simulations.

The main characteristics of circular motions by pigeon flocks are a highly synchronous state with unpredictable changes in rotational direction (Fig. 5A). As shown in Fig. 5B–D, another unique feature is that pigeons cyclically rotate around their loft during the flight. When a collective decision is made to change rotational direction, the average positions corresponding to three continuous instants lie on a relatively straight line. Thus, the radius of the curvature suddenly increases and hence the distance increases between the average circular center and the loft. To investigate more deeply into the mechanism underneath these behaviors, as shown in Fig. 5E–H, where we compare the results of the numerical simulations with the experimental data based on the trajectory containing both counter-clockwise and clockwise circular motion patterns. The numerical results agree well with the experimental data. More precisely, in the natural situation, pigeons fly in a three-dimensional space, so staggered overlaps are often observed along the two-dimensional projected trajectory. Therefore, we do not consider repulsion forces in the simulation. Analogously, when driven by the three forces in the present model, the individuals move collectively in circles with different radii. In addition, all of the pigeons change their rotational direction spontaneously, but never stray far from their loft.

## Discussion

Intermittent interaction exists in many species, such as locust^40^, sheep^41^, and humans^42^, among which Bazazi *et al*.^40^ reported that in the long movements which account for faster, and more energetic circling motions in the arena, intermittence can allow for some energy recovery. In this study, based on investigation of the circular motions, we suggest that pigeon flocks employ an intermittent interaction mechanism with low communication costs. In particular, each pigeon communicates with others only occasionally rather than continuously. The present study focuses on highly ordered circular motions, because turning information is propagated through the entire flock on almost every occasion, where the alignment of velocity fluctuations may carry significant directional information of interactions^35, 43^. Clearly, coordinated flock spatial structure with a high correlation of pairwise velocities is the foremost fascinating result, yielded by inter-individual interactions, or conversely, interactions are ciphered in the spatial structure^12^. Such interactions may refer to Newtonian forces, visual/auditory signal guidances, and coordinated velocity alignment, etc^44^.

In a previous empirical study^35^, interaction was found consistent with the directional correlations quantifying the intensity of velocity alignment, i.e., ${C}_{ij}=\langle {s}_{i},{s}_{j}\rangle$, where *s*_*i*_ denotes the normalized velocity. Further, a unique index *J* was introduced as the strength in the interaction $\frac{J}{2}\langle {s}_{i},{s}_{j}\rangle$. Still, in another work^43^, to quantify the size of the 3d-domains, the correlation function of velocity fluctuations was defined as $C(r)=\frac{1}{{c}_{0}}\frac{{\sum }_{ij}{\overrightarrow{u}}_{i}\cdot {\overrightarrow{u}}_{j}\delta (r-{r}_{ij})}{{\sum }_{ij}\delta (r-{r}_{ij})}$, where *δ*(*r* − *r*_*ij*_) is a smoothed Dirac *δ*–function characterizing the pairs of birds at mutual distance *r*, *c*_0_ is a normalized factor, and *u*_*i*_ and *u*_*j*_ are velocity fluctuations of birds *i* and *j*, respectively. Clearly, a large value of *C*(*r*) implies that the vectors are nearly parallel and thus strongly correlated. They further suggested that each bird with an effective perception has a much larger correlation range than the direct inter-individual interaction range. Therefore, it is reasonable to believe that with a sufficiently high threshold of correlation, the alignment mechanism can be captured. In this study, it should be noted that the so-called interactions only correspond to the mutual alignment of velocity fluctuations for the entire flock to keep synchronous. Accordingly, pairwise alignment can result in the velocity correlation, and in turn, such correlation may imply the causal velocity alignment as well. We conduct a numerical study based on the standard Vicsek model, which indicates that the instantaneous pairwise velocity alignment can be effective to reveal the underlying interaction among coordinated clustered entities (Figure S9). Limited to the current available data, it still remains a mystery what the real interaction is in natural bird flocks. It is hard to answer whether the time delay between two correlated individuals may imply the real leader-follower relationship, or it is just caused by the response to a third focal individual or even more complicated situations. These questions leave the door open to the future research on collective animal behaviors.

Furthermore, synchronous organization based on a jointly connected communication protocol substantially reduces the intra-group communication cost. Unlike human social networks, pigeons are not likely to establish connections with fixed neighbors^45^. Moreover, the interaction network of a pigeon flock is even almost always unconnected at each instant, whereas it becomes connected when all the networks are merged over sufficiently long consecutive time intervals. Thus, pigeon flocks employ a jointly connected principle instead to achieve coordination, thereby substantially reducing the communication costs, which better explains actual intermittent communication situations in biological groups^5, 46^. Significantly, the study provides biological evidence of the existence of jointly connected communication network in bird flocks.

The jointly connected condition guarantees that each individual can communicate with others directly or indirectly after a sufficiently long time. However, what will happen if an agent escapes the influence of the others for a long period? In the present numerical simulation, by using a weaker jointly connected condition with a faster decay rate of *ρ*, an individual far from the others becomes an outlier after a change in the current movement pattern, so it whirls constantly and spontaneously as observed in nature^17^. Although Ferdinandy *et al*.^47^ suggested that a centrifugal or centripetal force is not needed to induce spontaneous rotation (a scattered distribution of individuals along the circle), if the animal group has incentive to stay in one place, we still introduce a centripetal force for each individual to achieve the cohesively circular movements, and the independent rotation. We suggest that to achieve collective coordination, the influence of alignment should be sufficiently strong, especially under some specific sensitive conditions, such as the rotational direction switching time and the occurrence of conflicting decisions. With respect to the way for pigeon flocks to achieve cohesion and synchronization, the revealed connectivity probability trend here provides an explanation. Note that unlike the previous study^21^ on the way of information transmission in starling flocks, the connections in the network correspond to the channels of pairwise interaction defined as the alignment of behaviors, but not the extremely swift information propagation. However, we suggest that to maintain cooperation, small-sized pigeon flocks employ a rapidly-increasing connectivity mechanism, where the decays of interaction possibility with the increasing of inter-individual distance is similar to the scale-free decays of correlation in huge bird flocks^43^. In addition, we suggest that instantaneous correlation method helps discover the interaction mechanism from a dynamical perspective in collective behaviors. Interaction occurrence rate also suggests another way to identify reciprocal pairwise relationship in hierarchical leadership networks.

In terms of energy savings in biological groups, previous studies on V-shaped flocks of geese^48, 49^ have been conducted from an aerodynamics perspective and claimed that individuals favor energy savings. However, Usherwood *et al*.^50^ stated that unlike V-formation pelicans, pigeons with increased flap frequency do not gain any aerodynamic advantage from flying in a flock. Still, other studies^5, 46^ have focused mainly on self-assembly shapes and formations, rather than the pairwise interaction protocols considered in the present study. In the scenario where a pigeon has no neighbors occupies a large proportion, we suggest that communication energy is substantially saved. In future investigations, it will be necessary to scale up from the current small flocks to larger ones, and to check the inter-specie issue to other kinds of animal groups like fish schools and insect colonies. A previous study^12^ of huge starling flocks indicated that each individual only interacts with a fixed limited number of topological neighbors, which supports the communication energy saving result. However, do larger groups of coordinated animals employ intermittent or frequent communication mechanisms? Whether their interaction manners obey strongly or weakly connected network structures? These appealing questions merit further investigation.

## Methods

### Velocity fluctuation

To investigate the influences of the velocity fluctuations of one pigeon on others, we calculate the derivation from the mean velocity for each pigeon *i*, which is defined as ${\overrightarrow{u}}_{i}(t)={\overrightarrow{v}}_{i}(t)-1/N{\sum }_{k\mathrm{=1}}^{N}{\overrightarrow{v}}_{k}(t\mathrm{).}$ The spatial mean of the velocity fluctuations is zero by construction, thereby implying zero net motion at the center of mass^43^.

### Instantaneous correlation

Considering that the maximum of time delay in flock *D* is around one second^17^, we accordingly formulate the quantity related to the instantaneous correlation of two pigeons with time delay −1*s* ≤ *τ* ≤ 1*s* as

1 $${P}_{ij}(t)=\mathop{max}\limits_{\tau }\{{U}_{ij}(t,\tau )\}\mathrm{.}$$

Therein, the instantaneous correlation of velocity fluctuations is defined as

2 $${U}_{ij}(t,\tau )=\frac{2\cdot {\overrightarrow{u}}_{i}(t)\cdot {\overrightarrow{u}}_{j}(t+\tau )}{|{\overrightarrow{u}}_{i}(t){|}^{2}+|{\overrightarrow{u}}_{j}(t+\tau ){|}^{2}}\mathrm{.}$$

Thus, ${U}_{ij}(t,\tau )\in [-1,\,1]$, and ${U}_{ij}(t,\tau )=\pm 1$ if ${\overrightarrow{u}}_{i}(t)=\pm {\overrightarrow{u}}_{j}(t+\tau )$. This instantaneous correlation (Eq. (1)) integrates the correlation of velocity fluctuations. A sufficiently high value of ${P}_{ij}\ge {P}^{\ast }=0.92$ corresponds to the situation where the velocity fluctuation correlation is strong. In this case, the two pigeons are considered to align with each other, and hence pigeon *i* has the neighbor of pigeon *j*. (Setting the threshold of *P*_*ij*_ is shown in Figure S10). It should be noted that due to the difficulty to detect the real interaction in natural animal groups, we adopt the well-accepted assumption^12, 35, 43^ that the high correlation of the velocity fluctuations from each pair of birds quantifying the alignment intensity implies the occurrence of interaction. The results based on different values of *P* ^*^ are shown in Figure S10, where the intermittent interaction can still be observed.

### Definitions of the forces

In the model, we consider a group of *n* = 10 units moving in a planar space, where each has a velocity in two-dimensional real space, i.e., ${\overrightarrow{v}}_{i}\in {{\mathbb{R}}}^{2}$. Furthermore, ${\overrightarrow{v}}_{i}:={[{v}_{0}\cos {\theta }_{i},{v}_{0}\sin {\theta }_{i}]}^{{\rm T}}$, ${v}_{i}=||{\overrightarrow{v}}_{i}||$, where *ν*_*i*_ and *θ*_*i*_ are the linear speed and direction of individual *i*, respectively. For conciseness, we omit the time variable *t* (e.g. *v*_*i*_ = *v*_*i*_(*t*)), which denotes the step in the simulation. Let ${\overrightarrow{p}}_{i}:={[{x}_{i},{y}_{i}]}^{{\rm T}}$ be the particle’s Cartesian coordinates. The centripetal force *F*_*cen*_ is a virtual force which will drive particle *i* to rotate independently. It is ciphered in the following dynamics of each individual *i*

3 $$\{\begin{array}{rcl}{\rm{d}}{\theta }_{i} & = & ({\omega }_{i}+{\eta }_{\omega }){\rm{d}}t,\\ {\rm{d}}{x}_{i} & = & ({v}_{0}+{\eta }_{v})\cos \,{\theta }_{i}{\rm{d}}t,\\ {\rm{d}}{y}_{i} & = & ({v}_{0}+{\eta }_{v})\sin \,{\theta }_{i}{\rm{d}}t,\end{array}$$

where *ω*_*i*_ is the angular speed which is fixed in the simulation, and *η*_*ν*_ and *η*_*ω*_ denote the random noise with values of ± 10 percentage of the linear and angular speeds, respectively.

Subsequently, the three types of forces are given through the following stochastic differential equation

4 $${\rm{d}}{v}_{i}=({F}_{{\rm{cen}}}+{F}_{{\rm{align}}}+{F}_{{\rm{home}}}){\rm{d}}t+\xi ,$$

with *ξ* denoting a Poisson process of delta-correlated white noise with zero mean, among which based on the empirical results that individuals interact with others by aligning their positions and moving angles, the alignment force *F*_align_ is derived from its neighbors in order to achieve the convergence of the motion centers. Thus, the alignment rule is defined as follows

5 $${F}_{{\rm{align}}}=-\sum _{j\in {N}_{i}}\alpha (\parallel {\overrightarrow{p}}_{ij}\parallel )[\cos \,{\theta }_{i}\,\sin \,{\theta }_{i}]{[f({x}_{ij})f({y}_{ij})]}^{{\rm T}},$$

with *i*, *j* ∈ {1, 2, …, *n*} and $\parallel {\overrightarrow{p}}_{ij}\parallel =\parallel {\overrightarrow{p}}_{i}-{\overrightarrow{p}}_{j}\parallel$ denoting the Euclidean distance between individual *i* and *j*. Analogously, *x*_*ij*_ = *x*_*ij*_ − *x*_*j*_ and *y*_*ij*_ = *y*_*i*_ − *y*_*j*_ denote the Euclidean distance between individuals *i* and *j* in *x*- and *y*-axes, respectively. It should be noted that the system dynamics is handled by discretization in the simulation. For counter-clockwise circular motion of the center of mass, the unit alignment forces *f*(*x*_*ij*_) and *f*(*y*_*ij*_) are defined as

6 $$\{\begin{array}{c}f({x}_{ij})={x}_{ij}-{r}_{i}\,\sin \,{\theta }_{i}+{r}_{j}\,\sin \,{\theta }_{j},\\ f({y}_{ij})={y}_{ij}+{r}_{i}\,\cos \,{\theta }_{i}-{r}_{j}\,\cos \,{\theta }_{j},\end{array}$$

with *r*_*i*_ = *v*_*i*_/*ω*_*i*_ denoting the radius of curvature, which contains the convergence rule of both individual positions and circle centers. By contrast, for clockwise circular motion of the center of mass, *f*(*x*_*ij*_) and *f*(*y*_*ij*_) are defined as

7 $$\{\begin{array}{c}f({x}_{ij})={x}_{ij}+{r}_{i}\,\sin \,{\theta }_{i}-{r}_{j}\,\sin \,{\theta }_{j},\\ f({y}_{ij})={y}_{ij}-{r}_{i}\,\cos \,{\theta }_{i}+{r}_{j}\,\cos \,{\theta }_{j}\mathrm{.}\end{array}$$

In the simulation, we pick

8 $$\alpha (x)=\{\begin{array}{c}{w}_{\alpha }\mathrm{(1}-x/\rho \mathrm{)\ \ \ \ 0}\le x\le \rho ,\\ \mathrm{0\ \ \ \ \ \ \ \ \ \ \ \ \ \ \ \ \ \ \ }x > \rho ,\end{array}$$

where *w*_*α*_ > 0 is the weight of alignment, and *ρ* denotes the range (Euclidean distance) of alignment to induce the intermittent interaction, which is defined as a triangular-wave function with constant positive and negative amplitudes *U*_*A*_, *U*_*B*_, and frequency *ρ*_*f*_. In particular, a larger value of *w*_*α*_ suggests a high rate of coordination.

The homing force denotes the attraction from the loft to each particle, i.e.,

9 $${F}_{{\rm{home}}}=\frac{1}{{c}_{0}}{(\frac{||{\overrightarrow{p}}_{i}-{\overrightarrow{p}}_{0}||}{L})}^{{w}_{\beta }},$$

where *c*_0_ is a constant, *w*_*β*_ denotes the strength of attraction, ${\overrightarrow{p}}_{0}:={[{x}_{0},{y}_{0}]}^{{\rm{{\rm T}}}}$ is the Cartesian coordinates of the loft, and *L* is the side length for the simulation space. The parameters in the simulation are shown in Table 1.

## Electronic supplementary material

Supplementary information
