# Supplementary material for: Intermittence and connectivity of interactions in pigeon flock flights
Source: Sci Rep. 2017 Sep 5;7:10452. doi: 10.1038/s41598-017-09986-5 (PMC5585366; doi:10.1038/s41598-017-09986-5)
Supplement: Supplementary file 1 — Supplementary information [file 41598_2017_9986_MOESM1_ESM.pdf]

Supporting Information of "Intermittence and connectivity of interactions in pigeon flock flights"

Duxin Chen[1,2,3], Xiaolu Liu[2], Bowen Xu[2], and Hai-Tao Zhang[2,*]

[1]*Guangdong HUST Industrial Technology Research Institute, Guangdong Province Key Lab of Digital Manufacturing Equipment, Dongguan, 523000, China*

[2]*Key Laboratory of Image Processing and Intelligent Control, School of Automation, State Key Laboratory of Digital Manufacturing Equipments and Technology, Huazhong University of Science and Technology, Wuhan, 430074, China*

[3]*Department of Biological Physics, Eötvös Loránd University, Budapest, H-1117, Hungary*

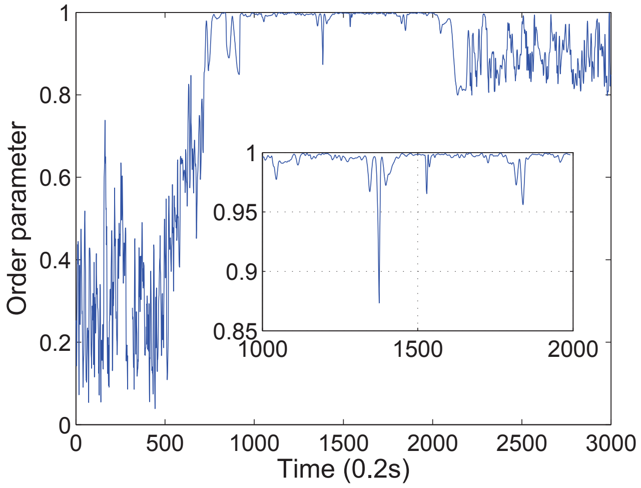

Figure S1: Evolution of the order parameter ϕ corresponding to the trajectory shown in Fig 5(A). To focus on the collective circular motions of pigeon flocks, we highlight the highly synchronous part with $\phi \geq 0.9$.

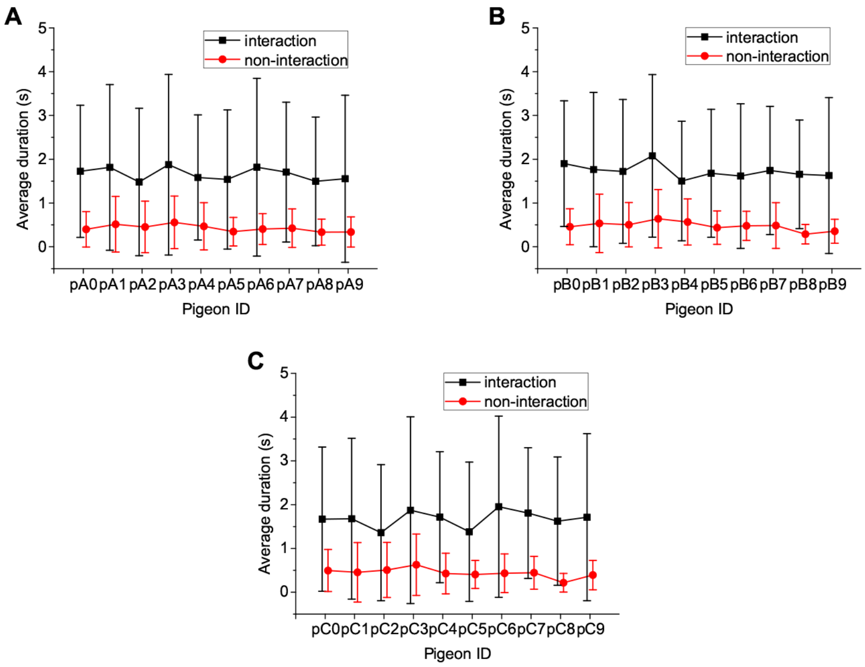

Figure S2: Average durations of both interaction and non-interaction situations corresponding to each pigeon over all the releases. Error bars indicate the standard deviations. For each individual, the average durations of both interaction and non-interaction situations remain steady, and the proportion of both situations quantifies the reduced communication energy, for (A) flock A, (B) flock B and (C) flock C.

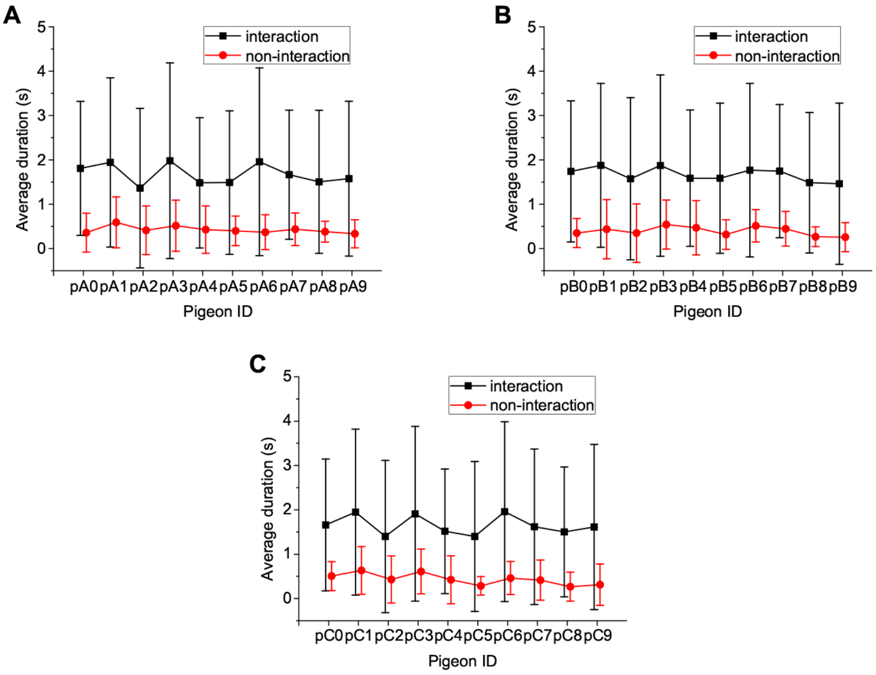

Figure S3: Average durations of both interaction and non-interaction situations for flocks A, B and C with sampling rate reducing to 5Hz. Error bars indicate the standard deviations. (A) Flock A. (B) Flock B. (C) Flock C. They also remain steady and keep consistent with the results for the data with sampling rate 10Hz, which verifies that sampling rate does not influence on the present results.

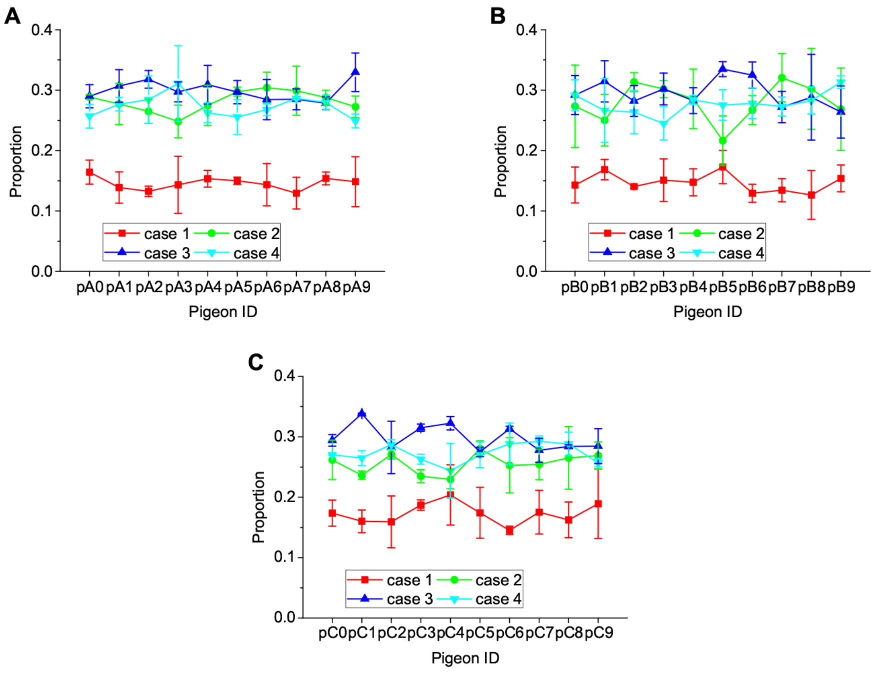

Figure S4: Proportions of the four cases in flocks A, B and C. *Case 1:* the pigeon only has dual interactions without time delay with other individuals; *Case 2:* the pigeon has directed interactions and plays the dual role as a leader and a follower simultaneously; *Case 3:* the pigeon has directed interactions and act as a follower; *Case 4:* the pigeon has directed interactions and plays the role as a leader. It is observed that *Case 1* where individuals only have undirected interactions without time delay occupies a smaller proportion, whereas directed interactions with time delay are more typical cases in pigeon flocks.

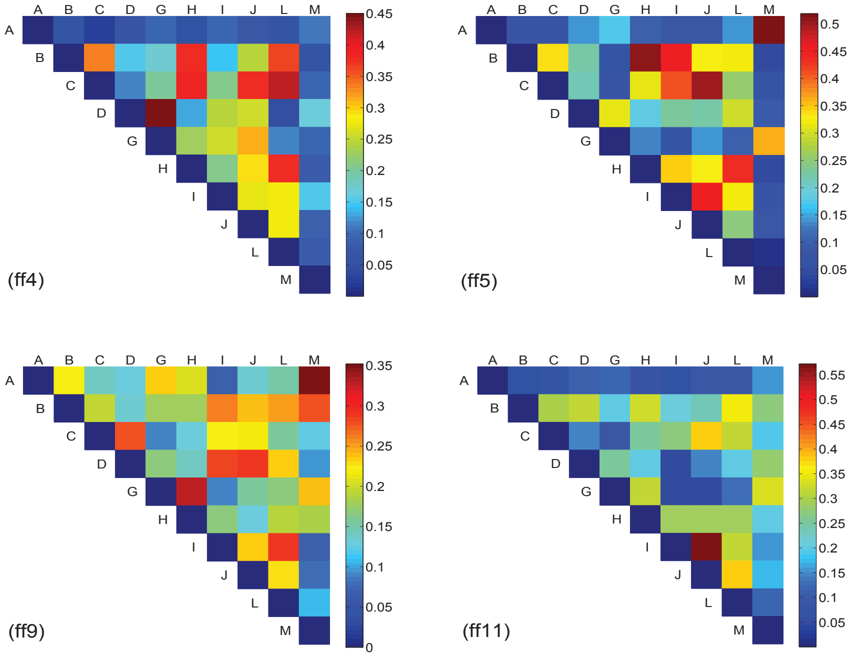

Figure S5: Interaction possibility of each pairwise members in flock D, releases ff4, ff5, ff9, and ff11. For the entire free flight, almost all members may have pairwise interactions among the flock. In addition, some pairs of pigeons may have more proportions than other pairs.

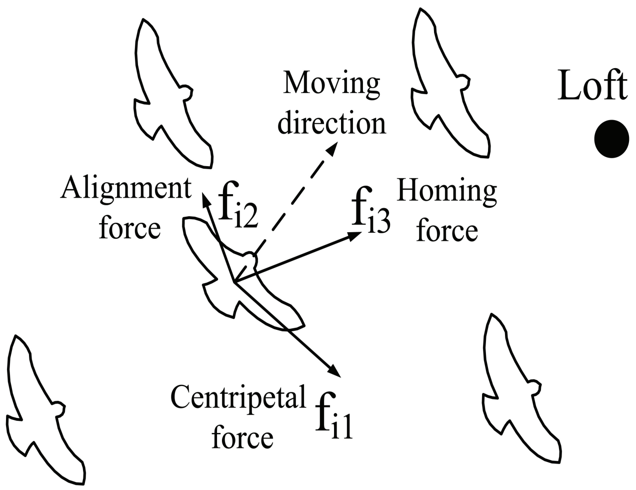

Figure S6: Illustration of three types of force act on a particle i. The centripetal force is designed to drive each individual to rotate. The alignment force is defined as the combined independent forces required from each neighbor in order to guarantee the convergence of the circular centers. The homing force denotes the attraction from the loft to each individual.

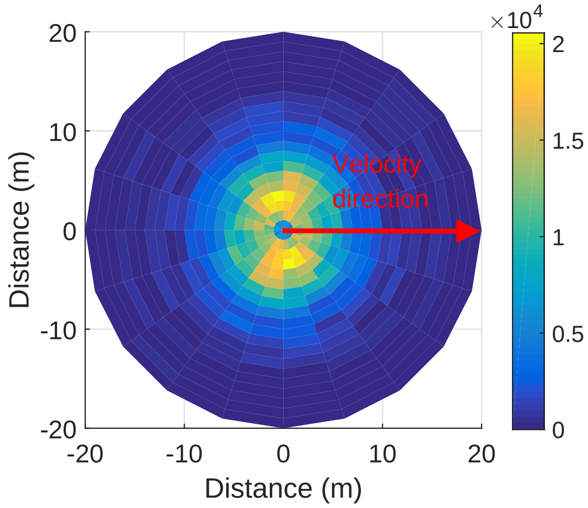

Figure S7: Distribution of interaction in flocks A, B, and C. The red arrow indicates the velocity direction, and distinct colors demonstrate different interaction frequencies. It is observed that individuals tend to interact with neighbors located right or left, but not along their velocity directions.

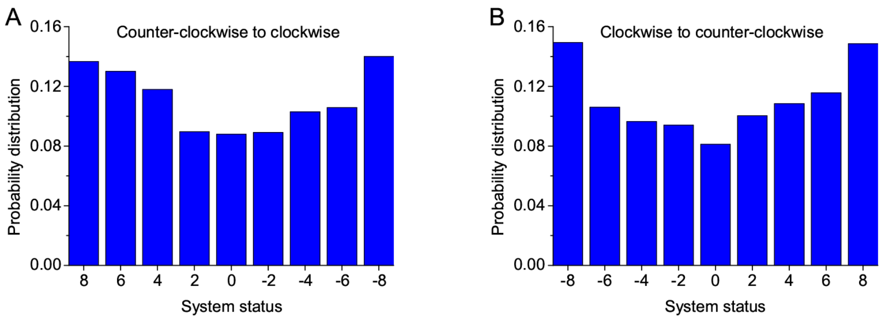

Figure S8: Probability distributions of the system status, i.e., the directional index of the entire flock. Here, "+1" and "−1" represent the individual counter-clockwise and clockwise rotations, respectively. With the reduction and increase of the index values, the entire flock gradually changes rotational direction from counter-clockwise to clockwise and the opposite, respectively. It is observed that the spontaneous changes of rotational directions follow a Gaussian distribution.

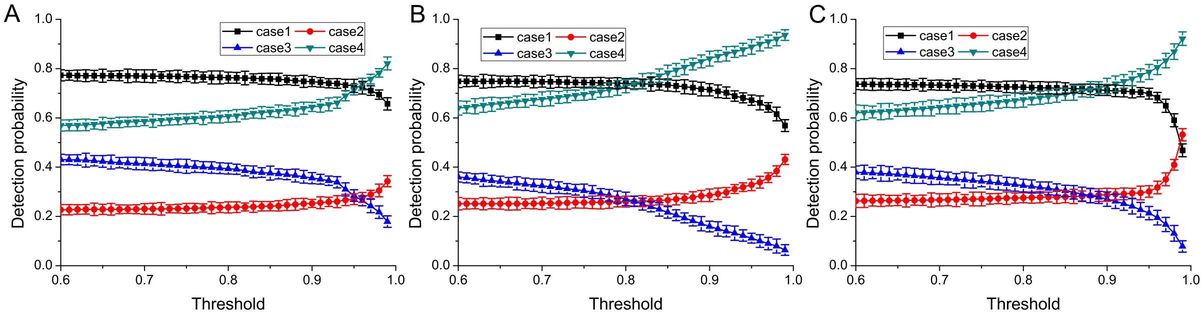

Figure S9: Correlation analysis on the standard Vicsek model (SVM). Therein, the parameters are set as $L = 50$, $N = 50$, $V = 0.2$, $M = 100$, which denote the size of the simulation space (i.e., Cartesian coordinates: $0 \sim 50$ in both x and y axes), the number of individuals, the preset speed, running steps, respectively. Note that simulations are independently run with one hundred times. Individuals are randomly initialized in the central area with Cartesian coordinates: $20 \sim 30$ in both x- and y- axes. (A) Simulation without noise, i.e., $\eta = 0$. (B) Simulation with a small magnitude of noise, i.e., $\eta = 0.01$. (C) Simulation with a big magnitude of noise, i.e., $\eta = 0.1$. Due to the interaction rules of SVM, we employ the instantaneous correlation function $C(t) = \frac{2*(u_i(t),u_j(t))}{u_i(t)^2+u_j(t)^2}$ to quantify the pairwise alignment without the index depicting time delay, where $u_i(t)$ denotes the velocity fluctuation of individual i at time t, i.e., $u_i(t) = v_i(t) - \sum_{i=1}^{N} v_i(t)$. With a threshold C^* used to distinguish the interaction or non-interaction situations, we can subsequently clarify the detection results into the four cases, i.e., Case1: the real interaction/alignment is correctly extracted; Case2: the real interaction/alignment is wrongly extracted; Case3: the non-interaction relationship is detected with wrong interaction; Case4: the non-interaction relationship is correctly detected. We observe that in the noise-free conditions, with the increasing value of C^*, either the real interaction/alignment or non-interaction relationship can be correctly detected with a high probability. Only if C^* is set excessively higher than 0.95, the detection probability of real interaction decreases, whereas the non-interaction relationship will be captured more exactly. The noise will not weaken the detection probability. Although the instantaneous correlation function quantifying the alignment intensity of velocity fluctuation cannot completely capture the real interaction or non-interaction relationship, the correct detection probabilities (both case1 and case2) are still sufficiently high. Thus, it may provide an effective way to detect interaction/non-interaction situations among coordinated groups.

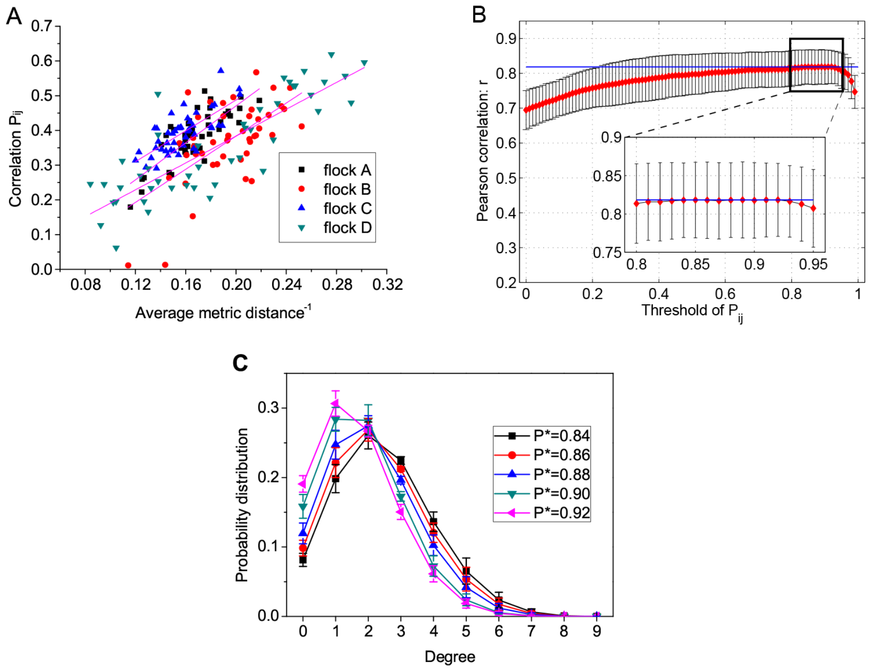

Figure S10: Setting the threshold of P_{ij}. (A) Scatter diagram for all possible pairs (45 pairs for 10 individuals) of each flock showing the relationship between the correlation function P_{ij} and the reciprocal of average metric distance. Significant positive correlation is observed for all flocks, which indicates that interaction and metric distance are correlated. Pearson's correlation tests: $r_A = 0.7950$, $r_B = 0.6153$, $r_C = 0.7363$, $r_D = 0.8455$, $n = 45$, $p < 0.001$. (B) r values of Pearson's correlation tests on the relationship between pairwise ratio of interaction and the reciprocal of average metric distance with different threshold in four flocks. Thus, the value 0.92 quantifying sufficiently strong positive correlation is selected as the threshold of P_{ij}. (C) Probability distribution of degree with different thresholds in four flocks. For highly ordered collective circular motions of pigeon flocks, difference in velocity fluctuations is not significant, and a higher threshold should be set to capture the situation that the pair of individuals are more synchronized. It is observed that with different thresholds (0.84–0.92), the intermittence of interaction still holds.
